# Error Analysis in Determination of Density and Temperature of Saline Solution Using Fiber Optic Photoacoustic Transducer Coated with MoS_2_-PDMS Composite

**DOI:** 10.3390/polym11050762

**Published:** 2019-05-01

**Authors:** Cheng Li, Jian Liu, Xiao Bin Peng

**Affiliations:** 1 School of Instrumentation Science and Opto-electronics Engineering, Beihang University, Beijing 100191, China; 18810927029@163.com (J.L.); 12171038@buaa.edu.cn (X.B.P.); 2 Shenzhen Institute of Beihang University, Shenzhen 518063, China

**Keywords:** fiber-tip photoacoustic transducer, MoS_2_-PDMS composite, density and temperature measurement, saline solution, error estimation

## Abstract

Regarding the ultrasound determination of density-dependent salinity in seawater, a miniature broadband (up to ~12.8 MHz at 6 dB bandwidth) fiber-tip photoacoustic transducer coated with an ~68.32 μm thick MoS_2_-polydimethylsiloxane (PDMS) composite was developed for simultaneously measuring the temperature and density of laboratory saline solutions, along with a piezoelectric transducer (PZT) for ultrasound detection. The two parameters, respectively, ranging 20 °C to 50 °C and from 0.99 g/cm^3^ to 1.10 g/cm^3^ were measured and then extracted based on the regressive dependence on the propagation speed and attenuation of the ultrasonic wave. In terms of the established linear regression model and estimated regression characteristic parameters, the calculated temperature and density results, respectively, exhibited the extended uncertainty values of 1 °C and 1.08 × 10^−3^ g/cm^3^ (*k* = 2.132), accompanied with an excellent goodness of fit (*R*^2^ > 0.97) and significance of the binary linear regression (*F* >> *F*_0.01_). The highly consistent experimental data confirmed the accuracy of our method, thus suggesting the potential of measuring salinity in seawater using compact fiber-optic photo-induced ultrasound scheme.

## 1. Introduction

It is well known that a great global conveyor belt runs deep under our oceans, which is powered by salinity that is a key variable in the modeling and observation of ocean water circulation, as well as the deep analysis of meteorological and oceanographic conditions [1,2]. Beginning in the 1950s, electrical conductivity measurements with sufficient accuracy were employed to detect the dissolved ionic content of seawater, which was then eventually established officially as the Practical Salinity Scale 1978 (PSS-78, UNESCO 1981a) [3], in which, however, a negative phenomenon was also mentioned that the relationship between solute mass and the electrical conductivity of real seawater may differ because of the composition differences, thereby appearing to be technically infeasible to reduce this uncertainty to a level that would satisfy oceanographic and climatological demands. In contrast, the relative uncertainty of seawater density measurements was about two orders in magnitude smaller than that in conductivity [3]. In fact, a new variable, the “Density Salinity” denoted by the symbol $S_{A}^{dens}$, is proposed as an observational parameter and represents the most appropriate representation of salinity for use in dynamical physical oceanography [4], wherein it is clearly further pointed out that the density salinity is the most useful to determine dynamical investigations of the ocean if a single salinity variable is required, although up to now no one salinity variable can fully characterize seawater with an anomalous composition. It is worth emphasizing that the most direct approach to determining the value of $S_{A}^{dens}$ for a particular water sample is to determine its density [4]. Moreover, a correlation equation to be used for the salinity calculation from density has been formulated as an IAPWS Release (IAPWS 2008) and is part of the Thermodynamic Equation of Seawater-2010 (TEOS-10). In this way, salinity can be readily available based on the measured density as a function of temperature, salinity and pressure, which has been increasingly concentrated on in previously reported literature [3,4,5,6,7]. Some typical experimental density data for seawater were also reported and summarized by Safarov et al. [8], wherein measurements of pressure, density and temperature of seawater with typical absolute salinity values were made based on the reproducibility of the density measurements by using a vibration-tube densimeter.

Hence, as a result of the disadvantages of a tedious and time consuming process for the direct determination of salinity, much effort has been made to perform the indirect measurements of salinity by the determination of density in laboratory saline solutions or seawater samples, which are usually conducted by measuring the electrical conductivity [9,10], refractive index [11,12], vibration frequency of resonant cantilever or tube [5,6,7,8,13] and ultrasonic properties [14,15]. Among these methods, significant differences in density measurement have been demonstrated when comparing the result of each approach. For example, conductivity, a measure of the ease of electrical current flow in solutions, reflects ion concentrations in solutions; however, the relationship is not straightforward and cannot account for all possible impurities [3,9]. Also, the geometry of a measurement cell containing a conducting electrolyte solution is required to be carefully determined by dimensional measurements to ensure the measurement uncertainty. Then a large amount of work has been advanced on the fluctuations in the refractive index of salt solution. However, due to limitations encountered when measuring the conditions of seawater, the spatial resolution of the obtained data is low, and the information about the inner scale is not available [16]. It is worthy to mention that currently the most commonly used densitometers are based on the principle of vibrating tubes (Coriolis flow meters), which can offer high precision density measurement. Unfortunately, these sensors are generally very time-consuming, require constant calibration and/or involve significant uncertainty about the origin of the sample used [14]. In addition, this method using a local vibrating probe is limited to pipe diameters below 60 mm; by contrast, an ultrasonic measurement can overcome this limitation, thereby allowing measurements in large pipes without pressure loss [17]. More importantly, compared to other techniques, ultrasonic measurement features with the advantages of being online, nondestructive and noninvasive and has competitive cost [18]. Nonetheless, it can be concluded from those previously reported works [14,15,17,18] that at present piezoelectric transducers (PZTs) are employed to generate ultrasound waves for density detection due to much higher signal-to-noise at a comparable bandwidth. Despite their excellent attributes, these sensors also suffer from several inherent disadvantages, such as electromagnetic interference (EMI) caused by electrical contacts, detection sensitivity restricted by the sensing element area and larger size [19]. In particular, fabrication of broadband electrical transducers with millimeter-scale lateral dimensions and integration into compact devices with meter-scale longitudinal dimensions can be challenging and expensive [20]. In contrast, miniature optical fiber ultrasound sources triggering can avoid those aforementioned limitations, in addition to featuring the intrinsic advantages such as flexible design, immunity to EMI and absence of electrical parts. Therefore, the choice of miniature fiber-optic ultrasound sources coated with sensitive photothermal materials is meaningful and necessary for simultaneous density and temperature photoacoustic (PA) measurement in solutions in consideration of the coupling effect of temperature. In reality, recently there has been rapid progress with the development of optical ultrasound transducers to address the limitations of electrical counterparts [19,20,21], but mostly they serve for PA biomedical imaging applications [22,23,24].

Hence in this paper, we develop a miniature optical fiber-tip ultrasound transmitter using a MoS_2_-PDMS composite coating to simultaneously measure density and temperature in laboratory saline solutions based on the PA effect, which contributes to investigating the determination of the density and then salinity of seawater in further research. Compared with recently reported gold nanostructures and graphene derivatives acting as light absorbers in previously fiber-optic PA transducers, MoS_2_ exhibits outstanding properties, such as a high absorbance coefficient in the near-infrared region (NIR), as well as good physiological stability and biocompatibility [25]. It is worth mentioning that to the best of our knowledge some efforts have been devoted to exploring the use of MoS_2_ currently concentrate on injecting photothermal materials into object samples; hence a complicated, large-size free-space PA microscopy is generally required, while with regard to characterizing solution properties, a miniature PA transducer coated with MoS_2_-PDMS composite has not yet been reported. Our experimental results show that the changes in velocity and attenuation of an ultrasonic wave, propagating in prepared saline solutions in the tested density range of 0.99–1.10 g/cm^3^ and temperature range of 20–50 °C, are extracted along with a good goodness of fit (*R*^2^ > 0.97) and significance of the linear regression (*F* >> *F*_0.01_), which implies a great potential for miniature and accurate ultrasound determination of salinity by measuring the density in seawater. Furthermore, the miniaturized coating dimension of ~68.32 μm × 125 μm (thickness × diameter) indicates that the developed optical fiber laser-generated ultrasound transducer could be a viable alternative to PZT ultrasound transducers in compact ultrasound detection.

## 2. Transducer Fabrication and Experimental Setup 

### 2.1. Fabrication of the Fiber-Tip PA Transducer

**Figure 1:**
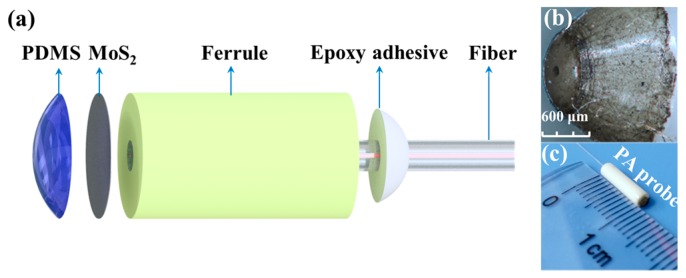
(**a**) Fabrication process of the developed PA transducer. (**b**) Microscope oblique view of the fiber-ferrule assembly coated with MoS_2_-PDMS composite. (**c**) Picture of the PA transducer.

Referring to the schematic fabrication process of the fiber-tip PA transducer as shown in Figure 1a, a commercial passive double-clad fiber (Passive-10/125DC, LIEKKI, Lohjac, Finland) with an outer cladding diameter of 125 μm was firstly inserted into a standard zirconia ferrule. Then a 1 μm resolution translation stage moved the double-clad fiber into the ferrule until their endfaces were aligned so that the fiber-ferrule assembly was secured by an epoxy adhesive. MoS_2_ dispersion liquid with a concentration of 1 mg/mL (www.xfnano.com) was extracted via a pipette and then was applied onto the fiber tip using drop casting. Note that since recently PDMS has shown great promise as an elastomeric polymer in the development of composite coatings for optical ultrasound generation due to its superior thermal expansion coefficient and high optical absorption [26], PDMS solution (Sylgard 184) was deposited on the MoS_2_-covered fiber tip through drop casting process. In this way, the outer diameter of the composite coating deposited on top of the outer cladding was considered as 125 μm. It is worth mentioning that before PDMS solidification, the composite coating-covered fiber-tip assembly was placed in a vacuum drying oven at 60 °C for about one hour to remove air bubbles in the cured MoS_2_-PDMS. After solidification, the composite was fixed to the endface of the fiber-tip as depicted in Figure 1b. It can be concluded from F-P interference measurement using an optical spectrum analyzer (AQ6370C, Yokogawa, Tokyo, Japan) that the developed probe exhibited a micro Fabry-Perot (F-P) structure whose the first mirror, the cavity and second mirror are the fiber-composite interface, the composite coating and the composite-air interface, respectively. Thus in terms of the demodulated F-P cavity length of ~68.32 μm, the thickness of the composite coating was approximated as the equivalent value. Figure 1c displays the fabricated PA probe. Our previous work can be referred to for more detailed information on preparing the MoS_2_-PDMS coating and transferring it onto the fiber tip [27].

### 2.2. PA Experimental Setup

**Figure 2:**
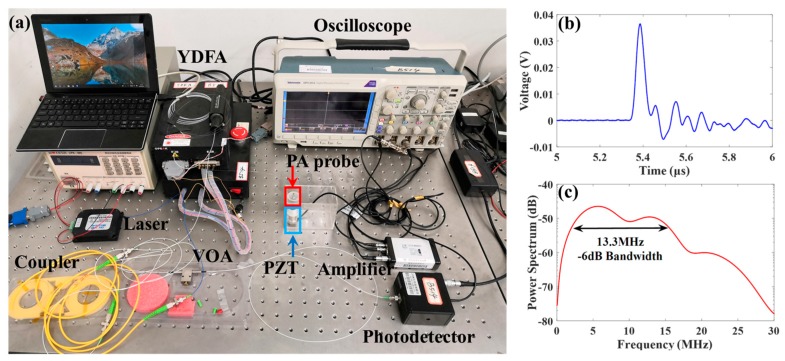
(**a**) Picture of experimental setup. Measured (**b**) time- and (**c**) frequency-domain PA pulse response of the developed transducer.

Figure 2a shows the experimental setup for density and temperature measurement in saline solution, wherein the developed PA transducer and a commercial 14.7 MHz PZT with a −6 dB bandwidth of 10 MHz for receiving ultrasound waves were respectively arranged on both endface sides of an acrylic glass tube with a dimension of Φ 16 mm × 40 mm (inner diameter × height) containing saline solutions. The distance between them was set as 7.7 mm by a vernier caliper with an accuracy of 0.1 mm. During experiment, the waterproof side of the tube fitted with the PZT would be placed in a water container for uniform water-bath heating. Then, a 5 ns, 1064 nm pulsed laser (OS-PL-M-5-5K-1064-10-1-S-FA, Beijing Conquer Optics Science & Technology Co., Ltd, Beijing, China) controlled by a trigger signal would generate a pulse excitation with a single pulse energy of 10 μJ to the probe through a 1 × 2 coupler after the power amplification via Ytterbium-doped fiber amplifier (YDFA). After the amplified pulsed light illuminated the core and inner cladding of the double-clad fiber, the PA signal induced by the photothermal effect of the composite coating was detected by the PZT receiver connected with its preamplifier (CTS-8682D, EasyNDT Co. Ltd, Shanghai, China). Meanwhile, another signal passing through the 1 × 2 coupler was captured by an oscilloscope (DPO3054) via a 200 MHz bandwidth photodetector through a variable optical attenuator (VOA). It is important to mention that although the laser damage threshold power value of the MoS_2_-PDMS composite has a marked impact on the signal and feasibility for use in miniature PA needles, the developed probe can offer a power density of ~31.85 mJ/cm^2^ in consideration of the cladding diameter of 125 µm for the used fiber, which is within the range of 2.5–87.9 mJ/cm^2^ previously reported for optical ultrasound generators using various PDMS composites [27]. PDMS is also available to raise the damage threshold of the ultrasound generators [28]; hence the laser fluence of ~31.85 mJ/cm^2^ is currently lower than the critical damage threshold of the MoS_2_-PDMS coating due to the intactness of the coating. The saline solutions are generally prepared for laboratory simplified experiments. Thus deionized water and table salt were sufficiently mixed via a glass rod and then ultrasonic cleaning to fabricate the target solution whose primary component is NaCl with a purity of more than 98%. The temperature and density of the prepared saline solutions were respectively confined to the range of 20–50 °C and 0.99–1.10 g/cm^3^. Note that the density as a function of temperature was typically chosen as 0.997, 1.006, 1.013, 1.019, 1.027, 1.034, 1.043, 1.052, 1.061, 1.069, 1.078, 1.087 and 1.098 g/cm^3^ at room temperature (24 °C). These density values were calibrated by a glass hydrometer with an accuracy of ±0.001 g/cm^3^ in advance. In this way, a total of 65 data combined by five temperature values and thirteen density values were finally set.

Then it can be clearly observed from Figure 2b that a high-amplitude direct ultrasonic wave appears at about 5.38 μs, which represents the generated PA response due to the optothermal effect of deposited PDMS elastomer under the short-pulse laser action. Figure 2c shows the extracted PA signal spectrum, which indicates a −6 dB bandwidth of around 13.3 MHz. Despite of the limited range, it is comparable to for those previously reported PA probes using gold nanocomposite with a bandwidth of ~8 MHz [19] and using CNT-PDMS coatings with a bandwidth of 12–15 MHz [20]. In reality, the power spectrum and bandwidth given in Figure 2c are also unavoidably affected by those of the PZT transducer. Hence an increased bandwidth is possible for the PA probe if a hydrophone with broader bandwidth is utilized. In this case, further research on composite coating structures and fabrication strategies is needed to enhance the bandwidth of the PA transducer.

## 3. Experimental Result and Analysis

**Figure 3:**
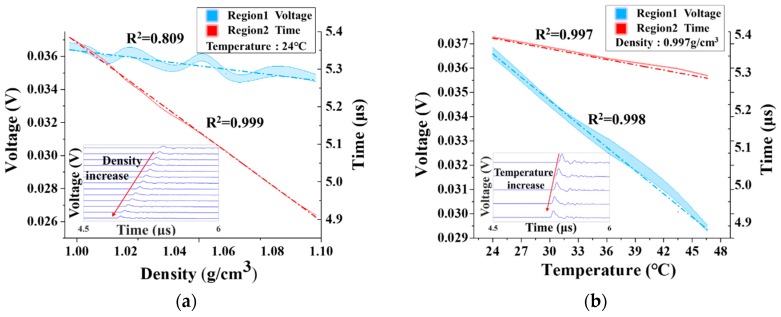
Measured PA signal in response to (**a**) *ρ* at 24 °C and (**b**) *T* at 0.997 g/cm^3^. Inset: Dependence of wave velocity on (**a**) *ρ* and (**b**) *T*.

The fabricated transducer’s PA response was examined by exposing it to the prepared saline solutions with various density (*ρ*) and temperature (*T*) values. The measurement in Figure 3a was repeated 5 times at a fixed temperature of 24 °C. In this case, the dependence of the measured voltage (*V*) and travelling time (*t*) on *ρ* was approximately fitted as 0.0179 V/g·cm^−3^ (*R*^2^ = 0.809) and 4.73 μs/g·cm^−3^ (*R*^2^ = 0.999), respectively, which are denoted by red and blue dashed lines by using least squares fitting method. The relation between *V* and *ρ* showed some weak nonlinearity. Then the standard deviations $s_{V,24{}^{\circ}C}$ and $s_{t,24{}^{\circ}C}$ of *V* and *t* at a certain density can both be solved by:

(1) $$s_{k,j\left(24{}^{\circ}C\right)}=\sqrt{\frac{\sum_{i=1}^{5}v_{i,k,j}^{2}}{n-1}},\left(k=V,t;j=1,\;2\ldots ,\;13\right),$$

where *v_i,k,j_* is the residual error of *V* or t at a certain density *ρ_j_* (*j* = 1, 2, …, 13). In this way, a total of 13 groups of standard deviation data were obtained, which represented the error variations of the two measured variables, labeled by red and blue regions in Figure 3a, whose average standard deviations were calculated as 0.23 ns and 0.24 mV, respectively. From the inset in Figure 3a, the travelling time of received PA signals decreased monotonically with the density, i.e., an increasing ultrasonic wave velocity, thus also indicating the possibility of accurate density measurement by linear regression.

Similarly, Figure 3b illustrates the dependence of *V* and *t* on *T* at a fixed density of 0.997 g/cm^3^, which were respectively fitted as 0.0003 V/°C (*R*^2^ = 0.998) and 0.004 μs/°C (*R*^2^ = 0.997) with an excellent goodness of fit (*R*^2^ > 0.99). The calculated error bands of the variables (*t* and *V*) with average standard deviations of 0.22 ns and 0.19 mV were marked by red and blue regions in Figure 3b. In like manner, both the small standard deviation and outstanding goodness of fit also further suggested the availability of accurate temperature measurement by linear regression. Thus, a monotonous increase in ultrasonic wave velocity with temperature occurred in the inset in Figure 3b. The fitted responses of 0.0179 V/g·cm^−3^ and 0.0003 V/°C revealed that a more remarkable linear impact on ultrasonic attenuation resulted from the temperature instead of the density. However, in view of 0.004 μs/°C and 4.73 μs/g·cm^−3^, the influence of the latter on wave velocity was approaching 4 times more than that of the former in the tested intervals of around 30 °C and 0.10 g/cm^3^.

**Figure 4:**
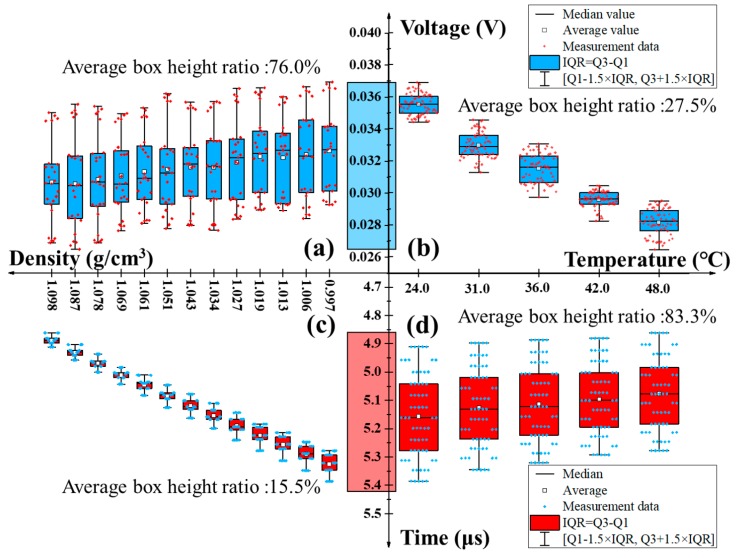
Boxplot of the measured *V* and *t* in response to various groups of temperatures and densities. Dependence of *V* upon (**a**) *ρ* and (**b**) *T* and dependence of *t* upon (**c**) *ρ* and (**d**) *T*.

In consideration of the density as a function of temperature, sixty-five data formed by five temperature values and thirteen density values mentioned above as a group were used for the following experiments where each measurement was repeated five times. Owing to the limited data (20 < *N* = 65 < 100), the IQR (inter-quartile range) was introduced so as to evaluate the error distribution of measured data [29]. Figure 4 displays the coupling variation in amplitude and velocity of ultrasonic wave with the temperature and density of saline solution in a form of a boxplot. Note that the boxplot is a standardized way of displaying the distribution of data based on a five number summary (“minimum”, first quartile (Q1), median, third quartile (Q3) and “maximum”), which can give a good indication of how the values in the measured data are spread out. In this way, all measurement data fell in the range of (Q1 − 1.5 × IQR, Q3 + 1.5 × IQR), where IQR = Q3 − Q1, and Q3 and Q1 are high and low quartiles of data samples, respectively. In other words, no gross error existed in the measured data. Then the comparison of box height determined by IQR in Figure 4a,b showed that the acoustic attenuation-dependent output voltage fluctuation caused by the temperature was generally about 3.52 times larger than that by the density, which could be obviously noticed from the rolling medians or average values. For example, the medians spanned from 0.0282 V to 0.0356 V in the range of 24–48 °C, while they only approximately changed by 2.1 mV in the range of 0.997–1.098 g/cm^3^, although a decreasing variation trend in amplitude was both achieved. Unfortunately, due to a limited number of temperature points, the box width induced by the former was relatively larger; however, this phenomenon may be easily overcome by reducing temperature intervals. In a same way, the comparison of box height determined by IQR in Figure 4c,d illustrated that the acoustic velocity-dependent travelling time fluctuation mainly resulted from by the density, which could also be obtained from the box height in the two figures. For instance, the box height in Figure 4c was within the range of 0.05–0.109 μs, whereas it increased to 0.411–0.475 μs in Figure 4d. More importantly, it can be seen from Figure 4c that the box height gradually decreased with the density. This can be explained by the fact that the effect of density on wave velocity is dominated, especially at higher densities.

Then in contrast to the average box height ratio (ratio of the difference between the upper and lower limits of a certain dependent output to the one between the maximum upper and minimum lower limits of all dependent outputs) of 76% among the corresponding output range in Figure 4a, Figure 4c exhibits a smaller average box height ratio of 15.5%. Likewise, Figure 4b has also a relatively lower average box height ratio of 27.5%, in comparison to that of 83.3% in Figure 4d. A lower ratio means more centralized data, therefore implying a higher degree of precision in density and temperature measurement by detecting the propagation speed and attenuation of ultrasonic wave in saline solution, respectively.

The correlation coefficient as an important parameter of evaluating the cross-coupling effect between any two of the independent variables (*T* and *ρ*) and dependent variables (*t* and *V*) is further illustrated in Table 1, where it calculated the Pearson correlation coefficients (*r*) of –0.018 and –0.955 between *T* and *t* or *V* by the use of Equation (2). The aforementioned negative sign (‘–’) indicated a reduction of *t* or *V* with the increase of *T*. Then the Pearson correlation coefficients between *ρ* and *t* or *V* were solved as −0.962 and −0.17, respectively. Among them, the larger r values (–0.955 and −0.962) close to 1 further verified the dominances of density and temperature upon acoustic propagation speed and wave attenuation, respectively. It should be mentioned that the Pearson correlation coefficient of −0.09 occurred between *T* and *ρ*, along with a significant of 0.104, which not only suggested a weak but non-significant correlation between them, but also accounted for the fact that the dependent variables *V* and *t* can be expressed only by the two independent variables *T* and *ρ*. That is to say, *T* and *ρ* of a certain saline solution are available to be exactly determined by measuring the amplitude voltage and travelling time of light-induced ultrasonic wave signal.

(2) $$r=\frac{\sum_{i=1}^{65}\left(X_{i}-\bar{X}\right)\left(Y_{i}-\bar{Y}\right)}{\sqrt{\sum_{i=1}^{65}{\left(X_{i}-\bar{X}\right)}^{2}}\sqrt{\sum_{i=1}^{65}{\left(Y_{i}-\bar{Y}\right)}^{2}}},$$

where *X* and *Y* are two different variables; $\bar{X}$ and $\bar{Y}$ represent the mean values of *X* and *Y*, respectively.

**Figure 5:**
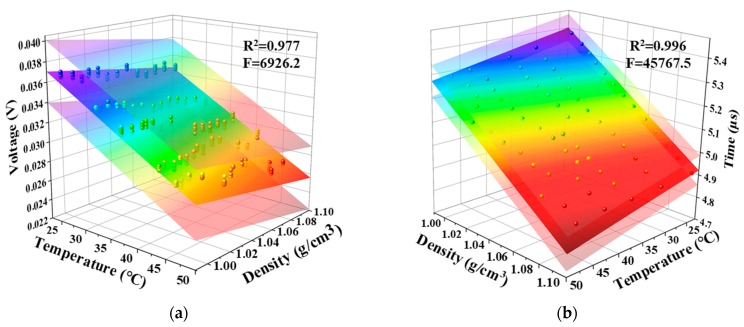
Binary linear regression models of *ρ* and *T* via measured (**a**) *V* and (**b**) *t*.

As thus, for the repeated five times of measurements (total 325 data points), the measured ultrasonic signal intensity *V* in saline solution as a function of *T* and *ρ* was confirmed by a binary linear regression model with an excellent goodness of fit (*R*^2^ = 0.977) and significance of the linear regression (*F* = 6926.2 >> *F*_0.01_ = 4.97) corresponding to the middle fitting surface in Figure 5a, which can be approximated as:

(3) $$V=-3.34\times 10^{-4}T-2.22\times 10^{-2}\rho +6.67\times 10^{-2},$$

In a similar way, Figure 5b presents the acoustic travelling time as a function of *T* and *ρ* with a similar high goodness of fit (*R*^2^ = 0.996) and significance of the linear regression (*F* = 45767.5 >> *F*_0.01_ = 4.97) corresponding to the middle fitting surface in the figure mentioned above, which can be given by:

(4) $$t=-4.73\times 10^{-3}T-4.37\rho +9.84,$$

It is important to point out that a large *F*-statistic value means a high significance of the whole fitted regression model instead of the regression coefficients in the model. Hence, a t-test was then adopted to evaluate the relevance of these coefficients. The t-test values (*t_bi_*) of three coefficients on the right side of equal sign in Equation (3) were in turn 115.95, 30.58 and 86.19 that are by far greater than *t*_0.01_(∞) = 2.63. For this reason, the dependency of both *T* and *ρ* on *V* was non-negligible. In the same way, the correlation effects of the two parameters on t are also prominent in Equation (4) due to the solved t-test values of 81.30, 297.57 and 629.18 greater than *t*_0.01_(∞) for the coefficients on the right side of equal sign. Thus the linearity between the regression coefficients and the dependent variables in Equations (3) and (4) was highly significant. However, in view of the incommensurability induced by units for these coefficients, the two equations after dimensionless standardization can be rewritten as:

(5) V=−0.98T−0.26ρ

and

(6) t=−0.27T−0.99ρ,

where the constant coefficients are called standard regression coefficients (or beta-weights) that are computed by dividing a parameter estimate by the ratio of the sample standard deviation of the dependent variable to the sample standard deviation of the regressor, as shown in Equation (7).

(7) $$b_{i}^{\prime }=b_{i}\frac{s_{X_{i}}}{s_{Y_{j}}},\;i=1,2;\;j=V,t,$$

where $b_{i}$ and $b_{i}^{\prime }$ (*i* = 1, 2) are the fitting coefficients of the independent variables (*T* and *ρ*) before and after standard regression, respectively; $s_{X_{i}}$ and $s_{Y_{j}}$ are the sample standard deviations of the dependent variable and the regressor variable, respectively. In this case, judging from the standardized regression coefficients in Equation (5), the most influencing factor was the temperature variable, which was ~3.77 times higher than the density. By contrast, it could be found that the coefficients in Equation (6) highlighted the dominated effect of the density variable, which was approximately 3.67 times greater than the temperature. The overall conclusions obtained by the standardized regression Equations (5) and (6) were in well agreement with the aforementioned results in Figure 3 and Figure 4.

**Figure 6:**
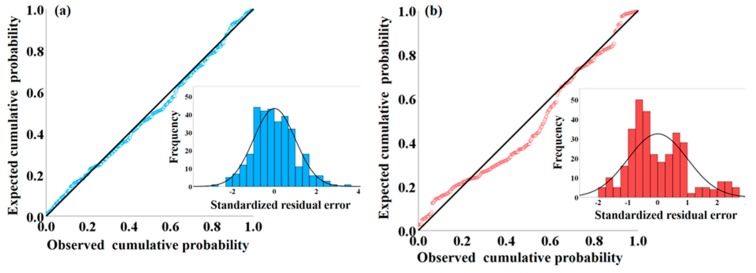
Normal P-P plot of regression standardized residuals of dependent variables (**a**) *V* and (**b**) *t*. Insets: Regression standardized residuals.

Although the coefficients in Equations (3) and (4) or Equations (5) and (6) characterized the significance of the regression models, the uncertainties of these coefficients were closely related to the scope of the established linear regression plane. Thus according to the t-test of coefficient significance, the 95% confidence interval (*α* = 0.05) on the fitting coefficients *b_i_* (*i* = 0, 1 and 2) corresponding to constant, temperature and density terms in Equations (3) and (4) is defined by [30]:

(8) $$\left[{\hat{b}}_{i}-t_{\alpha /2}\times s_{{\hat{b}}_{i}},\;{\hat{b}}_{i}+t_{\alpha /2}\times s_{{\hat{b}}_{i}}\right],$$

where ${\hat{b}}_{i}$ is the least squares estimate of *b_i_*; $s_{{\hat{b}}_{i}}$ is the standard deviation of ${\hat{b}}_{i}$. The limits on the dependence of *V* or *t* on *T* and *ρ* were denoted by the shadow regions in Figure 5a,b, respectively, wherein the residual errors in output voltage and wave travelling time characterized by the limits were further estimated in the following Figure 6. Furthermore, for comparative analysis, the regression characteristic parameters mentioned above were summarized in Table 2.

It should be noted that random errors often have a Gaussian normal distribution primarily because the normal distribution often describes the actual distribution of the random errors in real-world processes reasonably well. The normal distribution is also the most established model to characterize quantitative variation of data [31]. In such case, we used normal probability- probability (P-P) plot for comparing the data from the standardized residuals of the linear regression model with the normal distribution, thus evaluating whether the error term is actually normally distributed, as shown in Figure 6. It can be seen clearly from Figure 6a that the observed cumulative probability agreed well with the expected cumulative probability with an exceedingly high goodness-of-fit based on P-P probability plots, which was represented by $k_{0}^{2}=0.996$ in terms of the measure of linearity for standardized P-P plots [32]. In other words, since the reference oblique line obeys standard normal distribution *N*(0, 1), the regression standardized residual of dependent variable *V* significantly follows the normal distribution, as illustrated in the inset in Figure 6a. In the same manner, the observed cumulative probability in Figure 6b was also in agreement with the expected cumulative probability with a good linearity of the pattern of points on the plot ($k_{0}^{2}=0.969$). The relatively obvious deviation can also be noticed in the inset in Figure 6b, wherein the number of residuals in the region of [−1, 0] surpasses the standard normal distribution. However, this part of residuals still lied in the region of [−2, 2] corresponding to the 95% confidence interval. Hence, with regard to the super high goodness of fit (*R*^2^ = 0.996) and significance of the binary linear regression (*F* = 45767.5) obtained in Equation (4), the regression standardized residual of dependent variable *t* also followed the normal distribution on the whole, on basis of the histogram and normal distribution curve formed on it in Figure 6b.

**Figure 7:**
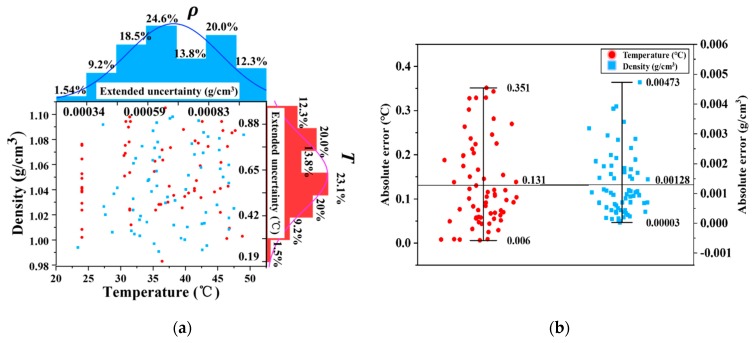
(**a**) Extended uncertainty and (**b**) absolute error distributions of resolved *T* and *ρ* values.

In order to evaluate the measurement performance of the proposed PA transducer, the relative and absolute error distributions of measured *T* and *ρ* relative to their reference values provided by the thermometer (testo 925) with an accuracy of ±(0.5 °C + 0.3% of measured value) and hydrometer are given in Figure 7. In Figure 7a, the extended uncertainties for total 65 groups of temperature and density data were determined with a coverage factor *k* (*k* = 2.132) on the basis of a confidence probability of 95%. Hence the maximum and minimum extended uncertainties for temperature were solved as 1 °C and 0.19 °C, respectively. In contrast, the maximum and minimum extended uncertainties for density were around 1.08 × 10^−3^ g/cm^3^ and 2.14 × 10^−4^ g/cm^3^. In fact, compared with the latter, the former displayed a more scattered temperature error distribution. For example, in a distribution form of relative error determined by the ratio of the regressor to the reference value, approximately 33.8% of the tested temperature data was within the relative error range of 0–0.2%, while nearly 80% of the tested density data fell in the same limit. It needs to be explained that the maximum error ranging from 0.2 °C to 0.25 °C at the upper limit temperature close to 50 °C is comparable with the uncertainty of ±0.5 °C induced by the used thermometer. Then as shown in Figure 7b, the maximum absolute errors of measured *T* and *ρ* were about 0.351 °C and 0.00473 g/cm^3^, respectively, along with standard deviations of about 0.094 °C and 0.0010 g/cm^3^. As mentioned above, regarding measurements of typical absolute salinity values based on the reproducibility of the density measurements by use of a vibration-tube densimeter, our density measurement results were compared with those using the aforementioned densimeter. Unfortunately, the former with a maximum standard uncertainty of 5 × 10^−4^ g/cm^3^ is currently still inferior to the one with a density measurement level of 10^−6^ g/cm^3^ for salt contents at atmospheric pressure in recently published work by Schmidt et al. [33]. The reason for the high measurement uncertainty relative to the high level of 10^−6^ possibly results from the temperature-induced thermal deformation of acrylic glass tube and composite coating, water evaporating and the inhomogeneity in salinity in salt solutions. The density in seawater therefore has not currently been measured with the present bare PA probe yet. In addition to the need of extremely low measurement uncertainty, actually the influence of seawater environmental parameters (in particular temperature and pressure) should also be considered [34], such as the laser wavelength shift and thermal expansion of composite coating caused by temperature, and the damping effect on elastic deformation of PDMS induced by increasing seawater pressure with depth increase. In this case, in addition to specific mechanical design and materials, further research on environmental variable compensation is needed to optimize the PA measurement uncertainty for achieving improved independence versus temperature and pressure variations, which contributes to ultimate higher precision density and salinity measurement. 

As a consequence, the proposed PA probe enables a potential ultrasound measurement for the temperature, density and resulting other parameters such as density-dependent salinity in seawater, in combination with the established regressive dependence upon the propagation speed and attenuation of ultrasonic wave. Furthermore, along with the proposed error evaluation scheme, the developed miniature fiber-optic ultrasound measurement could be further extended for the use of ultrasonic exposimetry in diagnostic ultrasound [35] and ultrasonic non-destructive testing [36].

## 4. Conclusions

A compact in situ fiber-optic ultrasound method using the PA effect excited by MoS_2_-PDMS composite coating deposited onto the fiber-tip endface was demonstrated to simultaneously perform the temperature and density measurement in saline solution in the tested range of 20–50 °C and 0.99–1.10 g/cm^3^, in order to investigate the promising potential for use of ultrasonic determination of the density-dependent salinity in seawater. The fabricated PA probe herein features with high NIR absorbance coefficient of MoS_2_ acting as the light absorber, in addition to miniaturized lateral dimension, simplified fabrication and ease of integration into existing compact measurement devices. On the basis of the fitted binary linear regressive models on the dependent variables (*V* and *t*) with an excellent goodness of fit (*R*^2^ > 0.97), significance of the binary linear regression (*F* >> *F*_0.01_) and high linearity of the pattern of points on the P-P plot ($k_{0}^{2}>0.96$), the measured temperature and density data followed a normal distribution with good measurement uncertainty for the repeated five times of measurements. Moreover, higher precision density and then salinity measurement is available if environmental variable compensation is needed to achieve improved independence versus temperature and pressure variations, thereby opening a potential PA approach to indirectly evaluate salinity by obtaining the density and its temperature effect in laboratory saline solutions or seawater.

## Figures and Tables

**Table 1:** Calculated Pearson correlation coefficients of the variables *T*, *ρ*, *t* and *V*.

Variable	Index	*T*	*ρ*	*t*	*V*
***T***	*r*	1	–0.090	–0.180 *	–0.955 * ^1^
	significance	/	0.104	0.002	<0.001
***ρ***	*r*	–0.090	1	–0.962 *	–0.170 *
	significance	0.104	/	<0.001	0.002
***t***	*r*	–0.180 *	–0.962 *	1	0.425 *
	significance	0.002	<0.001	/	<0.001
***V***	*r*	–0.955 *	–0.170 *	0.425 *	1
	significance	<0.001	0.002	<0.001	/

^1^ The values with the symbol ‘*’ represent the significant correlation at 0.01 level.

**Table 2:** Calculated regression characteristic parameters.

Equation		*R* ^2^	*F*	*b_i_*	$b_{i}^{\prime }$	*t*(*b_i_*)	*b_i_* Confidence Interval (95%)	
							Lower	Upper
(3)	*T*	0.977	6926.2	−3.34 × 10^−4^	−9.98	115.95	−3.34 × 10^−4^	−3.29 × 10^−4^
	*ρ*			−2.22 × 10^−2^	−0.26	30.58	−2.37 × 10^−2^	−2.08 × 10^−2^
	*C* ^1^			6.67 × 10^−2^	/	86.19	6.52 × 10^−2^	6.83 ×10^−2^
(4)	*T*	0.996	45767.5	−4.73 × 10^−3^	−0.27	81.30	−4.85 × 10^−3^	−4.62 × 10^−3^
	*ρ*			−4.37	−0.99	297.57	−4.40	−4.34
	*C* ^1^			9.84	/	629.18	9.81	9.87

^1^ The symbol ‘*C*’ represents the constant term in Equations (3) and (4).
